# Effectiveness and safety of anlotinib with or without S-1 in the treatment of patients with advanced hepatocellular carcinoma in a Chinese population: a prospective, phase 2 study

**DOI:** 10.2478/raon-2023-0036

**Published:** 2023-07-26

**Authors:** Mafei Kang, Feng Xue, Shengyuan Xu, Jieqiong Shi, Yunyan Mo

**Affiliations:** Department of Medical Oncology, Affiliated Hospital of Guilin Medical University, Guangxi Guilin, China

**Keywords:** hepatocellular carcinoma (HCC), anlotinib, S-1, molecule targeted therapy, chemotherapy

## Abstract

**Background:**

The aim of the study was to observe the safety and efficacy of anlotinib (ANL) alone or combined with S-1 in the first-line treatment of advanced hepatocellular carcinoma (HCC).

**Patients and methods:**

Fifty-four patients with untreated advanced HCC who could not be resected were randomly divided into the ANL group (n = 27) and ANL+S-1 group (n = 27). The ANL group was given 10 mg ANL orally once a day for 14 consecutive days, stopped for 1 week, and repeated every 21 days. The ANL+S-1 group was given 10 mg ANL once a day orally and 40 mg S-1 twice a day orally for 14 consecutive days, stopped for 1 week, repeated every 21 days. All patients were treated until the disease progressed or toxicity became unacceptable. For patients who could not tolerate adverse reactions, the ANL dose should be reduced to 8 mg per day. CT or MRI was reviewed every 6 weeks to evaluate the efficacy.

**Results:**

A total of 44 patients were included in the results analysis, including 22 patients in the ANL group and 22 patients in the ANL+S-1 group. In the ANL group, the objective response rate (ORR) was 4.5% (1/22), the disease control rate (DCR) was 77.3% (17/22), the median progression-free survival (PFS) was 4.2 months (95% CI: 3.6–6.0) and the median overall survival (mOS) was 7.0 months (95% CI: 6.3–9.0). In the ANL+S-1 group, the ORR was 18.2% (4/22), the DCR was 59.1% (13/22), the median PFS was 4.0 months (95% CI: 3.6–5.4) and the mOS was 6.0 months (95% CI: 5.5–7.4). There was no significant difference in ORR (*p* = 0.345) or DCR (*p* = 0.195) between the two groups. Adverse reactions were mainly hypertension, anorexia, fatigue, liver transaminase heightened and hand and foot skin reaction.

**Conclusions:**

ANL monotherapy was effective in the treatment of advanced HCC, and adverse reactions have been able to tolerated.

## Introduction

Hepatocellular carcinoma (HCC) is the most common type of primary liver cancer, accounting for 90% of primary liver cancers. China has a high incidence of HCC, accounting for more than half of the new cases in the world each year, and ranks second only to lung cancer in tumor-related deaths.

Common treatments for HCC include tumor resection, liver transplantation, transarterial chemoembolization, radiotherapy and molecular targeted drug therapy.^1^ In China, 70%–80% of patients have advanced stage or distant metastasis at the time of clinical first diagnosis, and the opportunity for surgical resection is lost. Transarterial chemoembolization (TACE) is the most commonly used method for the treatment of advanced HCC, but it is difficult to completely block the blood supply around the tumor.^2^ Therefore, drug therapy (cytotoxic drugs, multitarget antiangiogenic drugs, immune checkpoint inhibitors, etc.) has become a very important comprehensive treatment for advanced HCC.

Sorafenib is the first drug approved for the treatment of HCC, and both the SHARP study and Oriental study have confirmed that sorafenib can significantly prolong the survival of patients with advanced HCC. In the SHARP study of Westerners, the median overall survival (mOS) of sorafenib alone was 10.7 months^3^, while in the Oriental study of Asians, the mOS of sorafenib alone was only 6.5 months.^4^ Chinese studies have shown that the mOS of sorafenib alone in the treatment of advanced HCC is 6.0 months, which is similar to that of Oriental patients, suggesting that the efficacy of sorafenib in the treatment of HCC is poor in Asian populations. Therefore, finding more effective drugs for the treatment of HCC is of great clinical significance.

Studies have shown that anlotinib (ANL) is effective and well tolerated as a treatment for patients with advanced HCC.^5,6^ The results showed that fluorouracil was effective for HCC.^7,8,9^ Both S1 and capecitabine are fluorouracils. S-1 does not cause hand-foot syndrome and is more reasonable to combine with ANL, which has the potential to cause hand-foot syndrome, so our study design used ANL plus S-1.

The purpose of this study was to explore the safety and efficacy of ANL alone or in combination with Tigio (S-1) in the treatment of advanced HCC.

## Patients and methods

### Study design and participants

This is a prospective, single-center, real-world study to evaluate the efficacy and safety of ANL with or without S-1 in the first-line treatment of patients with advanced HCC. From February 2019 to August 2021, 54 HCC patients with Barcelona Clinic Liver Cancer (BCLC) stage C (stage I–II, Child‒Pugh A–B, and at least one criteria: PS1-2 or vascular invasion/extrahepatic spread)^10^ confirmed by histopathological examination or in accordance with the clinical diagnostic criteria of primary liver cancer were included. This study was conducted in accordance with the Helsinki Declaration and approved by the Review Committee of the Ethics Committee of the affiliated Hospital of Guilin Medical University.

The main inclusion criteria were as follows: (1) Age ≥ 18 years old, BCLC stage C. (2) Eastern Cooperative Oncology Group (ECOG) physical status score: 0–2, and the estimated survival time was at least 3 months. (3) At least one measurable lesion evaluated using Response Evaluation Criteria in Solid Tumors version 1.1 (RECIST1·1). (4) Hemoglobin ≥ 95 g/L, leukocytes ≥ 4.0 × 10^9^/L and platelets ≥ 100 × 10^9^/L. Serum creatinine was normal, and ALT and AST were less than 2.5 times the normal upper limit. (5) There was no hypertension. (6) The patient signed the informed consent form. The main exclusion criteria were as follows: (1) A history of gastrointestinal bleeding or a clear tendency of gastrointestinal bleeding in the past 6 months, such as esophageal varices with the risk of bleeding, local active ulcer lesions, and fecal occult blood ≥ +. (2) Routine urine tests showed urinary protein ≥ + + or confirmed that 24-hour urinary protein was more than 1.0 g. (3) Patients with hypertension who could not be reduced to the normal range by antihypertensive drugs (systolic blood pressure > 140 mmHg, diastolic blood pressure > 90 mmHg).

### Procedures

The patients were randomly divided into the ANL group (n = 27) and the ANL+S-1 group (n = 27). Patients in the ANL group were treated with 10 mg of ANL once a day for 14 days. The ANL+S-1 group was treated with 10 mg of ANL once a day and 40 mg of S-1 twice a day. Both drugs were taken continuously for 14 days, discontinued for one week and repeated every 21 days. The two groups were treated with drugs until the disease progressed or could not tolerate adverse reactions. For patients who could not tolerate adverse reactions, the dose of ANL was reduced to 8 mg daily. The curative effect was evaluated by magnetic resonance imaging (MRI) or computed tomography (CT) every 6 weeks.

### Statistical analysis

The chi-square test was used to compare the counting data of the two groups, the Kaplan‒Meier method was used to generate a survival curve, and the log-rank test was used to compare the difference in PFS and OS between the two groups. We used SPSS software (version 25.0) to perform all the statistical analyses. All statistical tests were bilateral tests, and p < 0.05 was statistically significant. This study was registered at the Chinese Clinical Trial Registry (chictr.org.cn), registration number: ChiCTR1900022129.

## Results

### Patient characteristics

Table 1 summarizes the baseline characteristics of the participants.

**TABLE 1.** Baseline characteristics of the ANL group and ANL+S-1 group were compared

	**Group ANL n=27**	**Group ANL+S-1 n=27**	**x^2^**	***P***
Gender n %				
Male	23(85.2)	22(81.5)	0.133	0.715
Female	4(14.8)	5(18.5)		
Age median	54	56		
ECOG PS n %			0.912	0.340
1	8(29.6)	5(18.5)		
2	19(70.4)	22(81.5)		
Child-Pugh, n %			0.078	0.780
A grade	10(37.0)	11(40.7)		
B grade ≤ 7	17(63.0)	16(59.3)		
Stages (BCLC), n %				
C	27(100.0)	27(100.0)		
AFP n %			0.318	0.573
AFP ≥ 400 ng/mL	16(59.3)	18(66.7)		
AFP < 400 ng/mL	11(40.7)	9(33.3)		
HBV DNA n %			1.421	0.233
≥ 1.0 × 10^3^ IU/mL	6(22.2)	10(37.0)		
< 1.0 × 10^3^ IU/mL	21(77.8)	17(63.0)		
PVTT n %			0.092	0.761
PV1–3	18(66.7)	16(59.3)		
PV4	2(7.4)	3(11.1)		
EHS n %	9(33.3)	7(25.9)	0.355	0.551

AFP = alfafetoprotein; ANL = anlotinib; BCLC = BCLC staging system; ECOG PS = Eastern Cooperative Oncology Group performance status; EHS = extrahepatic spread; HPV = hepatitis B virus; PVTT = portal vein tumor thrombosis; VP1 = PVTT extending distal to the second portal branch; VP2 = PVTT extending to the second portal branch; VP3 = PVTT extending to the first portal branch; VP4 = PVTT extending to the main portal trunk or opposite-side portal branch

### Antitumor activity

**FIGURE 1.**
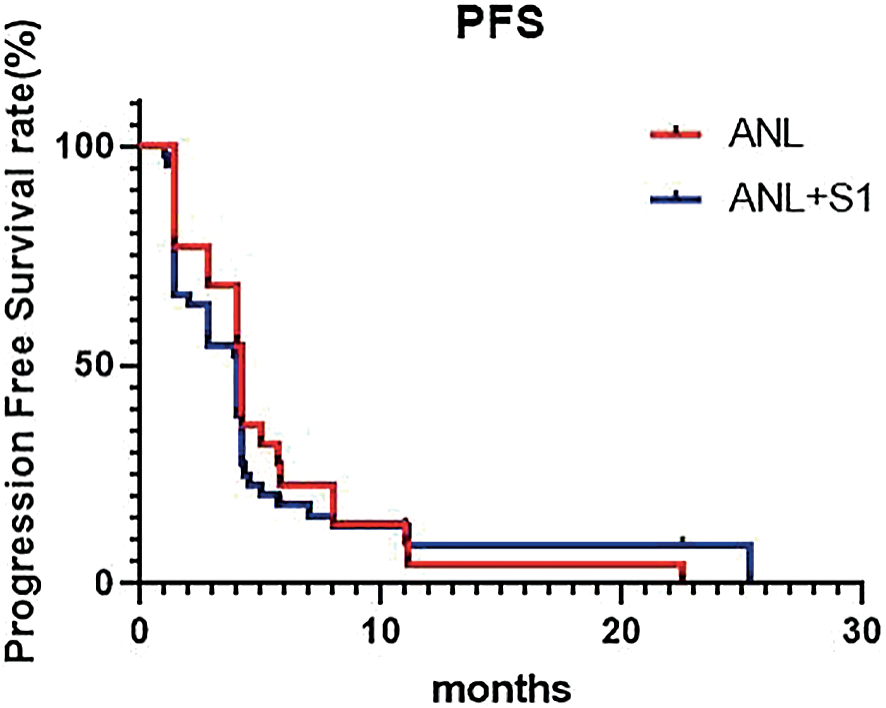
Comparison of progression-free survival (PFS) between the anlotinib (ANL) group and ANL+S1 group.

**FIGURE 2.**
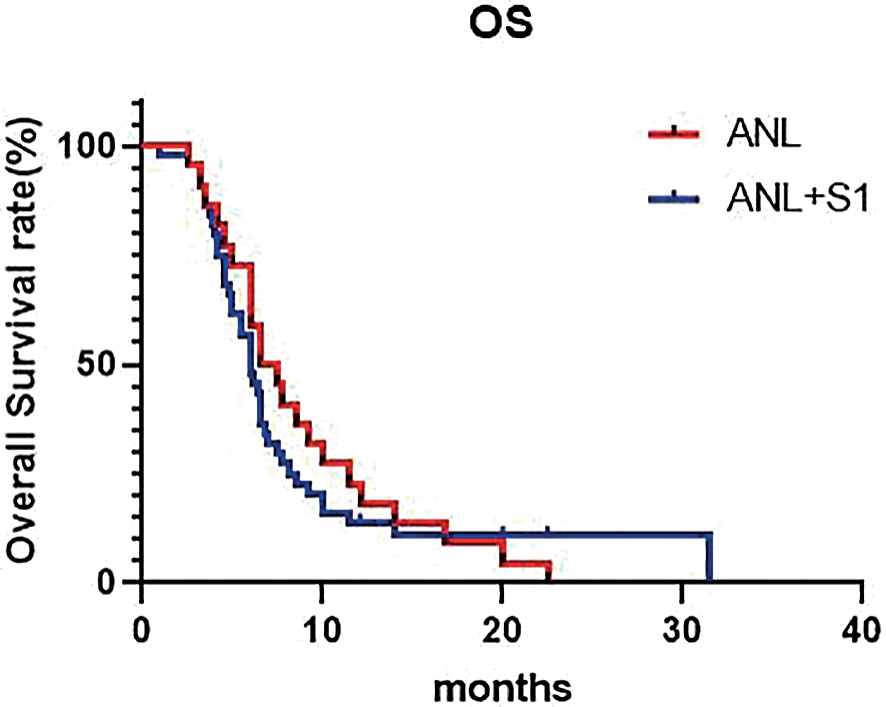
Comparison of overall survival (OS) between the anlotinib (ANL) group and the ANL+ S1 group.

A total of 44 patients were involved in the efficacy analysis (efficacy-evaluable population), with 22 cases in the ANL group and 22 cases in the ANL+S-1 group. In the ANL group, 1 (4.5%) patient achieved a PR, but none achieved a CR. The ORR was 4.5% (1/22), and the DCR was 77.3% (17/22). The median PFS was 4.2 months (95% CI, 3.6–6.0) (Figure 1, 2). The median OS was 7.0 months (95% CI, 6.3–9.0). In the ANL+S-1 group, 4 (18.2%) patients achieved a PR, but none achieved a CR. The ORR was 18.2% (4/22), and the DCR was 59.1% (13/22). The median PFS was 4.0 months (95% CI, 3.6–5.4) (Figure 1, 2). The median OS was 6.0 months (95% CI, 5.5–7.4). There was no significant difference in ORR (*p* = 0.345) or DCR (*p* = 0.195) between the two groups. The longest observation time in this study was 30 months, and the 1- and 2-year survival rates were 22.7% and 4.5% (ANL group) and 4.5% and 0.0% (ANL+S-1 group), respectively. Fisher's accuracy test revealed no significant difference in the 1-year and 2-year survival rates between the two groups. (P > 0.05).

### Safety

The most frequent adverse effects (AEs) were hypertension and fatigue. Hypertension, hand-foot skin, and diarrhea were among the grade 3 treatment-related AEs that occurred in 7 (15.9%) of the patients. Most of these events can be reversed by adjusting the dose of ANL or by taking other drugs (such as antihypertensive drugs). In the ANL group, 4 patients reduced the dose of ANL due to grade 3 or 4 adverse reactions. In the ANL+S-1 group, 3 patients reduced the dose of ANL due to grade 3 or 4 adverse reactions. There were no treatment-related fatalities. The treatment-related AEs did not interrupt the study. Table 2 shows all AEs, whether treatment-related or not.

**TABLE 2.** Incidence and grade of major adverse reactions in the ANL group and ANL+ S-1 group

**Adverse reactions**	**Group ANL (n=22)**				**Group ANL+S-1 (n=22)**				**x^2^**	***P***

	**Any level**	**1**	**2**	**3/4**	**Any level**	**1**	**2**	**3/4**		
Hypertension	11(50.0%)	7	2	2	12(54.5%)	6	3	3	0.910	0.763
Anorexia	5(22.7%)	4	1	0	8(36.4%)	5	3	0	0.983	0.322
Fatigue	16(72.7%)	14	2	0	18(81.8%)	16	2	0	0.518	0.472
Hand-foot-skin reaction	5(22.7%)	3	1	1	7(31.8%)	4	3	0	0.458	0.498
Leucopenia	3(13.6%)	2	1	0	4(18.2%)	3	1	0	0.170	0.680
Bleeding	1(4.5%)	0	1	0	0(0.0%)	0	0	0	0.410	0.235
ALT abnormal	6(27.3%)	6	0	0	8(36.4%)	7	1	0	0.419	0.517
Oral mucositis	3(13.6%)	1	2	0	5(22.7%)	3	2	0	0.617	0.432
Hypothyroidism	6(27.3%)	4	2	0	6(27.3%)	5	1	0	0.000	1.000
Diarrhea	1(4.5%)	0	0	1	2(9.1%)	1	1	0	0.364	0.546

The comparison of adverse reactions was the comparison of any grade data between the ANL and ANL+S-1 groups.

ALT = alanine transaminase; ANL = anlotinib

## Discussion

In this phase II prospective clinical trial, we observed the efficacy and safety of ANL alone or in combination with S-1 in patients with advanced HCC. To the best of our knowledge, this is the first controlled study to evaluate ANL alone or in combination with S-1 in patients with advanced HCC. The results showed that ANL had certain antitumor activity in the treatment of advanced HCC and that adverse reactions could be controlled. However, it would be necessary to compare ANL with standard treatment in a randomized trial with more patients included before this drug appears in standard practice for HCC.

Anlotinib is a novel multitarget tyrosine kinase inhibitor that mainly inhibits vascular endothelial growth factor receptor 2 and 3 (VEGFR2/3), fibroblast growth factor 1–4 (FGFR1-4), platelet-derived growth factor receptor α and β (PDGFR α/β), C-Kit and Ret.^11^ VEGFR, FGFR and PDGFR are related to tumor angiogenesis and growth. C-Kit and Ret are important members of the tyrosine kinase receptor protein family and receptors of stem cell factors. Their products are tyrosine kinase type 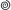, which makes tumor cells proliferate. Therefore, ANL could inhibit tumor cell and tumor vascular growth at the same time. Pharmacokinetic evaluation showed that ANL had a long elimination half-life (116 ± 47 hours) and significant accumulation after multiple oral administrations. In basic research on lung cancer, ANL induced apoptosis and protective autophagy in lung cancer cell lines. Autophagy inhibition further enhanced the cytotoxicity of ANL and enhanced the antiangiogenic effect of ANL through JAK2/STAT3/VEGFA signaling.^13^ ANL could inhibit the proliferation, migration and invasion of small cell lung cancer H446 cells by inhibiting the c-Met pathway and activating the ERK1/2 pathway.^14^ ANL can also induce apoptosis and inhibit proliferation of hepatocellular carcinoma cells through Erk and Akt pathways.^15^ The above studies showed that ANL inhibits tumor angiogenesis and promotes tumor cell apoptosis through multiple signal transduction pathways. Other studies have found that ANL overcomes the multidrug resistance of colorectal cancer cells to cytotoxic drugs by inhibiting the PI3K/AKT pathway^16^, suggesting that after treatment, patients are resistant to multiple cytotoxic drugs, and the combined use of ANL may be beneficial again.

The mechanism of action of ANL is similar to that of apatinib.^17^ Some studies have confirmed that the ORR of apatinib in the first-line treatment of HCC was 16%, the DCR was 60%, the PFS was 5 months and the OS was 13 months.^18^ From clinical practice, it was observed that the adverse reactions of ANL were lighter than those of apatinib, so we chose to use ANL in this study. S1 is fluorouracil. The results showed that fluorouracil was effective for HCC.^7,8,9^ S-1 has no adverse reaction to hand-foot syndrome, and it was more reasonable to combine with ANL, which may have hand-foot syndrome. Therefore, to achieve a better tumor control rate, longer survival time and better quality of life, we used ANL combined with S-1 for advanced HCC. Some studies have shown that the combination of ANL and the S-1 regimen was beneficial in the third-line treatment of non-small cell lung cancer, the OS of patients in the combination group was longer than that in the S-1 group^19,20^, and the ORR in the combined group was higher than that in the ANL group.^21^ However, the results of this study show that there is no significant difference in ORR, DCR, PFS and OS between the ANL group and the ANL plus S-1 group, indicating that there was no significant clinical benefit from the combination of ANL and S-1 in the treatment of advanced HCC, and whether it is related to the lower dose of S-1 needs further study. The results of this study showed that the mPFS and mOS of patients treated with ANL alone were 4.2 months and 7.0 months, respectively, of which 1 patient still had no progress after 22.5 months. For patients with BCLC stage C, the clinical benefits are positive. Clinical studies have shown that HCC patients with portal vein tumor thrombus (PVTT) can be treated with hepatic arterial infusion chemotherapy (HAIC) or sorafenib. Compared with VP4 (PVTT extending to the main portal trunk or opposite-side portal branch) patients, the OS of VP2 (PVTT extending to the second portal branch) – 3 (PVTT extending to the first portal branch) patients was 7.1 months and 5.5 months, respectively.^22^ Most of the patients in this study were PVTT patients, most of whom were VP1 (PVTT extending distal to the second portal branch)-3 type. The mOS of ANL alone was 7.0 months in our study, which was similar to that of sorafenib. In our study, there was no significant difference in the 1-year and 2-year survival rates between the two groups, suggesting that ANL combined with S-1 was no better than ANL alone.

In this study, the main adverse reactions included hypertension, loss of appetite, fatigue, increased liver transaminase and hand and foot skin reactions, most of which were grade 1–2, and only a few patients with grade 3–4 adverse reactions needed to reduce the dose of ANL, indicating that the side effects of ANL were tolerable. Adverse reactions in NSCLC patients treated with ANL included hypertension (67.4%), hand and foot syndrome (43.9%), hemoptysis (14.0%), elevated thyroid stimulating hormone (TSH) (46.6%) and corrected QT interval (26.2%).^23^ Our study showed that the incidence of the above adverse reactions was lower than that reported in the literature, which was related to the use of medium-dose ANL. The results of the ALTER-0303 study of ANL in the treatment of NSCLC showed that a total of 57.8% of patients received antihypertensive drugs for hypertension, 18.0% of patients received levothyroxine for hypothyroidism, 8.2% of patients received beta ester for high triglycerides, 3.7% of patients received cortisone cream for hand and foot syndrome, and 12.9% of patients received antidiarrheal drugs. In the ANL group, 8.16% and 10.54% of the patients needed to reduce the dose and stop taking drugs, respectively.^24^ Most small molecular inhibitors showed greater side effects due to the low selectivity of VEGFR2 tyrosine kinase, while ANL was a highly selective VEGFR2 inhibitor with fewer side effects. In this study, there were more patients with leukopenia in the ANL+S-1 group than in the ANL group, but there was no significant difference between the two groups, which may be related to the small sample size or low myelosuppression with a low dose of S-1. Studies have shown that grade 3 or more adverse events of daily 12 mg ANL treatment for liver cancer include hypertension (12.73%), decreased white blood cell count (3.64%), decreased absolute neutrophil count (1.82%), decreased platelet count (9.09%), fatigue (3.64%), decreased hemoglobin (1.82%) and diarrhea (1.82%). There were also phase II clinical studies that showed that the grade 3–5 adverse events of daily 12 mg ANL in the treatment of liver cancer were hypertension (8%), diarrhea (8%) and hand and foot syndrome (6%).^26^ In our study, the adverse reactions above grade 3 in the ANL group were hypertension (9.10%), diarrhea (4.55%) and hand and foot skin reactions (4.55%). There were no other adverse reactions above grade 3, and there were fewer adverse reactions above grade 3, which was considered to be related to the daily dose of 10 mg in this study.

The study has some limitations. First, this was a single-center study. Second, the study had a small sample size, with only 22 patients per cohort. The study is not powered enough to compare the ANL and ANL+S1 groups and to conclude that S1 does not add to efficacy.

## Conclusions

In conclusion, ANL monotherapy is effective in the treatment of advanced HCC, and adverse reactions can be tolerated. However, ANL combined with S-1 did not improve ORR and DCR or prolong PFS and OS in advanced HCC patients.
